# The feasibility study on the generalization of deep learning dose prediction model for volumetric modulated arc therapy of cervical cancer

**DOI:** 10.1002/acm2.13583

**Published:** 2022-03-09

**Authors:** Zhang Qilin, Bao Peng, Qu Ang, Jiang Weijuan, Jiang Ping, Zhuang Hongqing, Dong Bin, Yang Ruijie

**Affiliations:** ^1^ Department of Radiation Oncology Peking University Third Hospital Beijing China; ^2^ Center for Data Science Academy for Advanced Interdisciplinary Studies Peking University Beijing China; ^3^ Beijing International Center for Mathematical Research Peking University Beijing China

**Keywords:** cervical cancer, deep learning, dose prediction, generalization

## Abstract

**Purpose:**

To develop a 3D‐Unet dose prediction model to predict the three‐dimensional dose distribution of volumetric modulated arc therapy (VMAT) for cervical cancer and test the dose prediction performance of the model in endometrial cancer to explore the feasibility of model generalization.

**Methods:**

One hundred and seventeen cases of cervical cancer and 20 cases of endometrial cancer treated with VMAT were used for the model training, validation, and test. The prescribed dose was 50.4 Gy in 28 fractions. Eight independent channels of contoured structures were input to the model, and the dose distribution was used as the output of the model. The 3D‐Unet prediction model was trained and validated on the training set (*n *= 86) and validation set (*n *= 11), respectively. Then the model was tested on the test set (*n *= 20) of cervical cancer and endometrial cancer, respectively. The results between clinical dose distribution and predicted dose distribution were compared in the following aspects: (a) the mean absolute error (MAE) within the body, (b) the Dice similarity coefficients (DSCs) under different isodose volumes, (c) the dosimetric indexes including the mean dose (*D*_mean_), the received dose of 2 cm^3^ (*D*_2cc)_, the percentage volume of receiving 40 Gy dose of organs‐at‐risk (*V*_40_), planning target volume (PTV) *D*_98%_, and homogeneity index (HI), (d) dose–volume histograms (DVHs).

**Results:**

The model can accurately predict the dose distribution of the VMAT plan for cervical cancer and endometrial cancer. The overall average MAE and maximum MAE for cervical cancer were 2.43 ± 3.17% and 3.16 ± 4.01% of the prescribed dose, respectively, and for endometrial cancer were 2.70 ± 3.54% and 3.85 ± 3.11%. The average DSCs under different isodose volumes is above 0.9. The predicted dosimetric indexes and DVHs are equivalent to the clinical dose for both cervical cancer and endometrial cancer, and there is no statistically significant difference.

**Conclusion:**

A 3D‐Unet dose prediction model was developed for VMAT of cervical cancer, which can predict the dose distribution accurately for cervical cancer. The model can also be generalized for endometrial cancer with good performance.

## 1 INTRODUCTION

Compared with intensity modulated radiation therapy (IMRT), the treatment time of volumetric modulated arc therapy (VMAT) is significantly shorter, and the dose distribution is equivalent or even better, so VMAT is becoming more and more extensively applied to the treatment of various tumors.^1^ However, the plan design still has the following problems. Firstly, the planner needs to iteratively adjust the planning target volume (PTV) and organs‐at‐risk (OARs) optimization objective in the plan optimization process to meet the clinical requirements, which is a time‐consuming process. Secondly, due to the dose–volume histograms (DVHs) and dose distribution that can be achieved for a specific patient is unknown before the plan is optimized, the optimization objective setting largely depends on the experience of the planner, which makes the final plan quality and consistency poor.^2^, ^3^, ^4^

In recent years, knowledge‐based planning (KBP) has been proposed to improve planning efficiency and plan quality.^5^, ^6^, ^7^, ^8^, ^9^ The current KBP method mainly has two branches. One is based on the DVHs prediction model,^10^, ^11^, ^12^, ^13^, ^14^ which uses prior patient databases of high‐quality treatment plans to establish a model to predict the DVHs of the patient's specific structures and provide an optimization objective to guide the following plan optimization. The commercial software representative of the above method is RapidPlan (Varian Medical Systems, Palo Alto, CA, USA). Although the method based on the DVHs prediction model has been proven to be effective in many studies, it cannot provide spatial dose distribution information. The other one is based on the method of three‐dimensional (3D) dose distribution prediction model,^8^, ^15^, ^16^, ^17^, ^18^, ^19^, ^20^ which uses the machine learning or deep learning network to automatically extract the structure and dose features in the plan to establish a dose prediction model.^21^, ^22^, ^23^, ^24^, ^25^

Shiraishi and Moore^8^ manually selected features as input to the model and developed a 3D dose prediction model based on artificial neural networks (ANN). The average prediction error for all voxels was less than 8%. The accuracy of the dose prediction model based on ANN usually depends on the selection of manual features. It is difficult to improve the accuracy of dose distribution prediction as the extraction process of manual features is complicated and tedious. It is attractive to develop an algorithm that can automatically extract features from the patient's contour structures to achieve a more accurate and effective prediction for 3D dose distribution. Deep learning, especially convolutional neural network (CNN), has developed rapidly in recent years and has been used in the field of medical images. Compared with the dose prediction model based on machine learning, the model based on deep learning can accurately predict the dose distribution of a specific patient without manually extracting features.^19^, ^21^, ^26^, ^27^ CNN is a common network in deep learning, which can automatically extract hierarchical features from medical images and predict dose distribution without manual operations. U‐net is a preferred deep learning network for dose prediction, and it has been used in the establishment of dose prediction models for different diseases in previous studies.^18^, ^19^ Nguyen et al.^19^ first used 2D‐Unet to establish a dose prediction model for predicting prostate cancer IMRT dose distribution. Six contours of critical structures and dose distribution of clinical plans were used as input for training to learn local and global features in their work. The results showed that the mean absolute error (MAE) of PTV was less than 2%, and the prediction error of OARs was less than 5%. Compared with previous ANN methods, the 2D‐Unet method provides better prediction performance. However, the biggest challenge of this 2D U‐Net method is that it predicts the 3D dose distribution on a slice‐by‐slice basis instead of real 3D volume prediction. Such predictions may cause uncertainty, especially at the edge of PTV. Based on the shortcomings of 2D‐Unet, 3D‐Unet is more widely used in the construction of dose prediction models. Zhou et al.^28^ tested a 3D‐Unet dose prediction model for the dose prediction effect of rectal cancer IMRT, and the prediction results were not much different from the clinical results, the overall MAE was 3.92 ± 4.16%, the mean Dice similarity coefficients (DSCs) was above 0.9 for most isodose volumes. Kajikawa et al.^29^ established a dose prediction model for the IMRT plan for prostate cancer based on 3D‐Unet and compared the results with the RapidPlan. The MAEs of the 3D‐Unet dose prediction model were within 3% and 5% for PTV and OARs, respectively, which is lower than the RapidPlan model. Their study revealed the potential of 3D‐Unet for dose distribution prediction. Looking back at previous studies, it can be found that although the deep learning model has shown high dose prediction accuracy, most of the existing studies are based on a single cancer for training and testing. However, it requires a large number of training data to train a cancer‐specific model. Collecting such data is a time‐consuming process for clinicians. Therefore, it is necessary to test the dose prediction effect of the model between different cancers to explore the generalization of the model between different cancer cases, which could expand the scope of the application of the model. Secondly, most studies focus on the IMRT plan. It is more meaningful to establish a dose prediction model for the VMAT plan due to the advantage of the VMAT delivery efficiency. Finally, to our knowledge, there is no report of deep learning for cervical cancer dose prediction. Therefore, the purpose of this study is to explore the generalization of the dose prediction model of the cervical cancer VMAT plan for endometrial cancer.

## 2 METHODS AND MATERIALS

### 2.1 Plan information

One hundred and seventeen cases of cervical cancer and 20 cases of endometrial cancer VMAT plans that have been clinically treated were retrospectively selected for this study. This study was approved by the Institutional Review Board (IRB). Eighty‐six cervical cancer cases were randomly selected as the training set, 11 cervical cancer cases were used as the validation set, and 20 cases of cervical cancer and 20 cases of endometrial cancer were used as the test set, respectively. Two arcs were used for all VMAT plans. The photon energy was 10 MV and the prescription dose was 50.4 Gy in 28 fractions. The dose calculation algorithm was an anisotropic analytical algorithm (AAA) and the calculation grid was 2.0 mm. The plan design process was as follows: before the plan was designed, patient image acquisition was carried out, all patients were fixed with a thermoplastic body mask, and the image was acquired using a CT simulator (Brilliance Big Bore, Philips Medical Systems) in the supine position. The scanning slice thickness was 5 mm. Clinicians performed clinical target volume (CTV) delineation according to relevant delineation guidelines, and then expanded CTV by 0.5 cm to form PTV. After the PTV was delineated, OARs delineation was performed. The OARs involved in this study were bladder, rectum, left and right femoral heads, colon, and small intestine. The clinicians conducted a second check on the delineation results to ensure that the delineation was accurate and consistent. After the above work was completed, the medical physicist designed the radiotherapy plan on Eclipse15.6 (Varian Medical Systems). After the plan was completed, the clinicians reviewed and approved the plans.

### 2.2 Data preprocessing

All plans were exported using DICOM‐RT format files in Eclipse 15.6, including structure files and dose files. Firstly, the image matrix containing the contoured structures in the structure file was registered with the dose distribution matrix. The contoured structures image matrix contains eight contours: body, bladder, rectum, PTV, left and right femoral heads, colon, and small intestine. These structures were input into the network as independent channels. Secondly, in order to accelerate the training of the model and reduce the calculation time, the 512 × 512 matrix size of contoured structures and the dose distribution matrix were down‐sampled to 256 × 256 first. During training phase, a 128 × 128 × 64 3D matrix was sampled for each patient.

### 2.3 Network structure

#### 2.3.1 3D‐Unet structure

**FIGURE 1:**
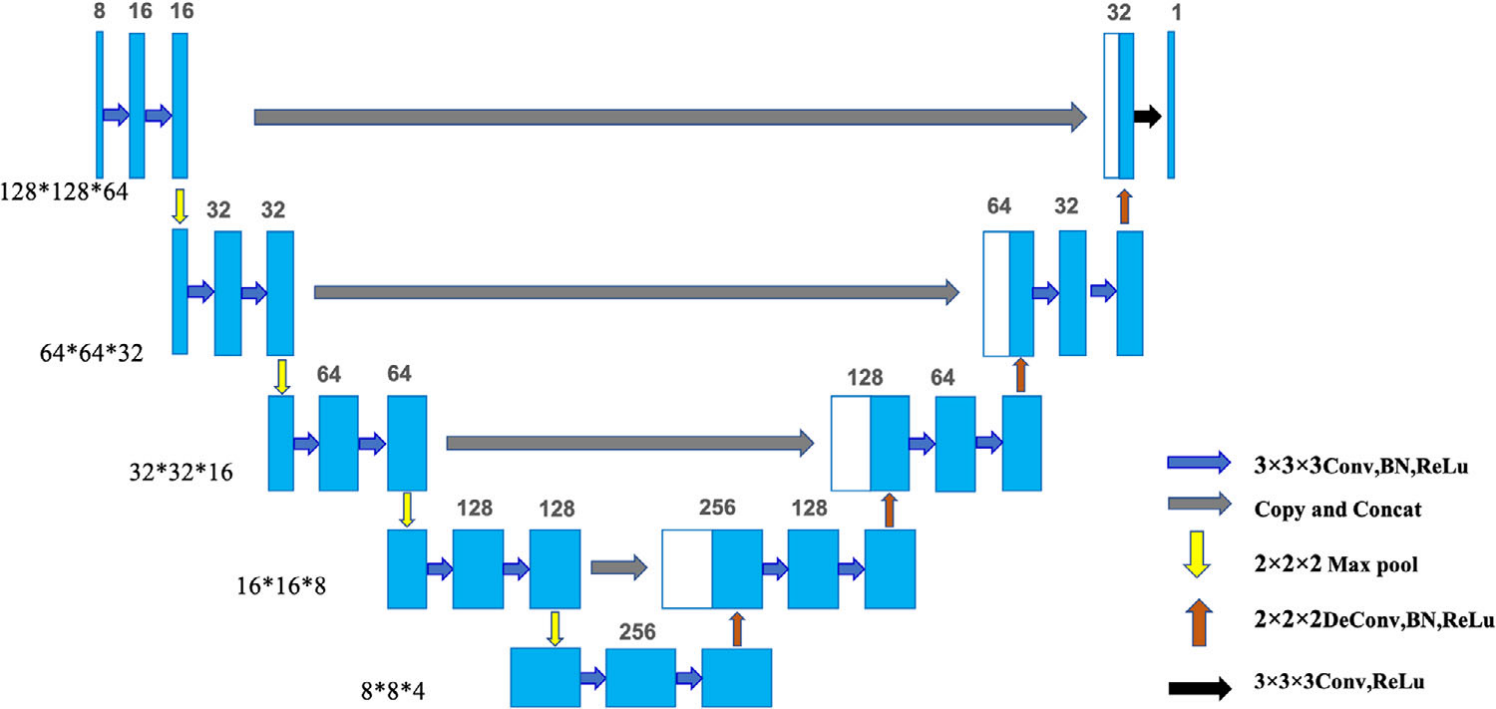
3D‐Unet architecture, the number on each box represents the number of features extracted

During training phase, the input was a 4D tensor with the size of 8 × 128 × 128 × 64 which consists of eight 128 × 128 × 64 contoured structures, and the output was a corresponding 3D dose distribution matrix with the size of 128 × 128 × 64. The network structure is shown in Figure 1. The network structure consists of three components: coding area, decoding area, and connection module. The connection module was used for layer jump connection and feature fusion. The dimensionality of the original data was reduced in the code area to extract more features. There were four code modules in the coding area. Each encoding module contained two 3 × 3 × 3 convolution layers and a 2 × 2 × 2 max‐pooling layer. After each convolution layer was a batch normalization (BN) and a rectified linear unit (ReLu). The decoding area also contained four decode modules. Each module contained two 3 × 3 × 3 convolutional layers and a 2 × 2 × 2 deconvolution layer. After each convolutional layer was also a BN and a ReLu. In the last layer, a single 3 × 3 × 3 convolution and ReLu activation function were used to output the final dose distribution matrix.

#### 2.3.2 Model training and validation

Eighty‐six patients were used as the training set and 11 patients as the validation set. The mean square error (MSE) was selected as the training loss function. The formula of MSE is as follows

$$\mathrm{MSE}=\frac{1}{n}\;\sum_{i\;=\;1}^{n}{\left(D_{p}^{i}-D_{c}^{i}\right)}^{2}$$

where *n* refers to the total number of voxels, $D_{p}^{i}$ refers to the predicted dose of the *i*th voxel, and $D_{c}^{i}$ refers to the clinical dose of the *i*th voxel. The adaptive moment estimation (Adam) algorithm was used to minimize the loss function. The initial learning rate was 10^–4^, and its decay rate of each epoch was the same as the decay rate of the cosine function during the training process. The python‐based PyTorch platform was used to build the 3D‐Unet deep learning architecture. Nvidia GeForce GTX 1080Ti GPU graphics card with 11 GB of memory was used for model training. After the model was trained, it only took a few seconds to predict the dose distribution for a specific patient from the test set. The network training curve is shown in supplement file.

#### 2.3.3 Evaluation of prediction results

The results between clinical dose distribution and predicted dose distribution of 20 cases of cervical cancer and 20 cases of endometrial cancer were compared.

- Comparison of 3D dose distribution

Firstly, the clinical dose distribution map, predicted dose distribution map, and dose difference map at different slices were compared in the test cases. Secondly, the MAE within the body in each of the 20 test cases were calculated. The formula is as follows:

$$\mathrm{MAE}\;=\frac{1}{n}\;\sum_{i}^{n}\left|D_{p}^{i}-D_{c}^{i}\right|,$$

where $D_{p}^{i}$ refers to the predicted dose of the ith voxel, $D_{c}^{i}$ refers to the clinical dose of the ith voxel, and n refers to the number of all voxels in the body. After calculating the MAE, it was divided by the prescribed dose and converted into a percentage. Thirdly, the DSCs of the different isodose volumes for each patient were calculated. In this study, the 10%–100% (interval of 10%) of the prescribed dose was selected as the isodose. The formula is as follows:

$$\mathrm{DSC}\;=\frac{2*V_{p}*V_{c}}{V_{p}+V_{c}}\;,$$

where $V_{p}$ represents the volume where the predicted dose distribution was greater than the given isodose and $V_{c}$ represents the volume where the clinical dose distribution was greater than the given isodose.

B. Comparison of dosimetric indexes and DVHs

1. Dosimetric indexes

For PTV, the homogeneity index (HI) and $D_{98\%}$ were calculated, the calculation formula for HI is as follows:

$$\mathrm{HI}\;=\frac{D_{2\%}-D_{98\%}}{D_{50\%}},$$

where *D_X_*_%_ represents the received dose of *X*% of the PTV volume. For OARs, the mean dose (*D*_mean_) and *V*_40_ (that is the percentage of the OARs volume that receives 40 Gy) of the bladder, colon, left and right femoral heads, rectum, and small intestine were evaluated. The received dose of 2 cm^3^ (*D*_2cc_) was also compared for the small intestine, colon, rectum, and bladder.

2. DVHs

The representative DVHs of 3D‐Unet model predicted dose distribution were compared with the clinical dose distribution for the tested cervical cancer and endometrial cancer, respectively.

## 3 RESULTS

### 3.1 Comparison of predicted dose distribution and clinical dose distribution

**FIGURE 2:**
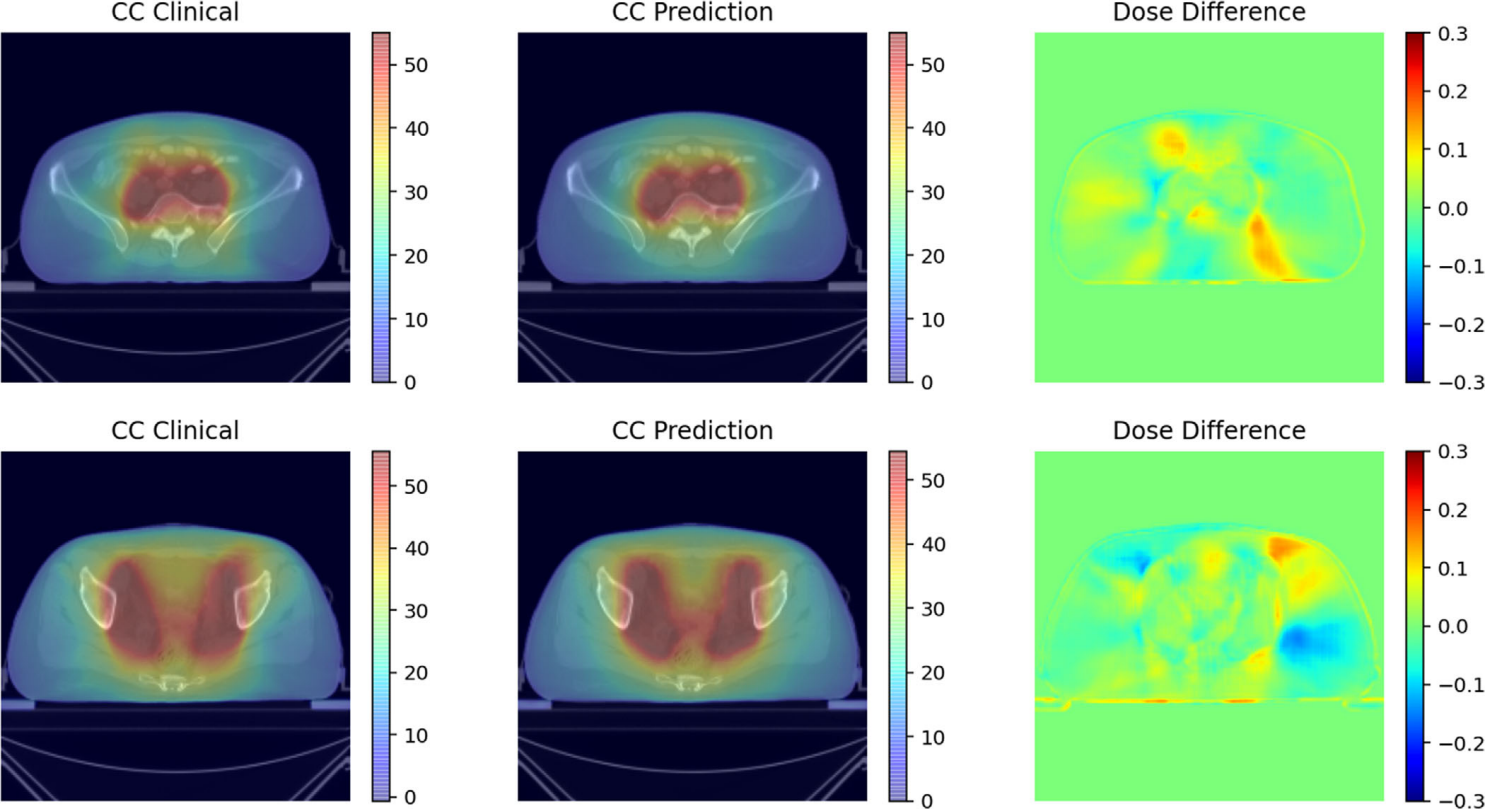
The clinical dose distribution map, the model predicted dose distribution map, and the dose difference map at the same slice of the cervical cancer case. The left side is the clinical dose distribution map (the unit is Gy), the middle is the predicted dose distribution map and the right is the dose distribution difference map (take the prescription dose equal to 1 as the standard)

**FIGURE 3:**
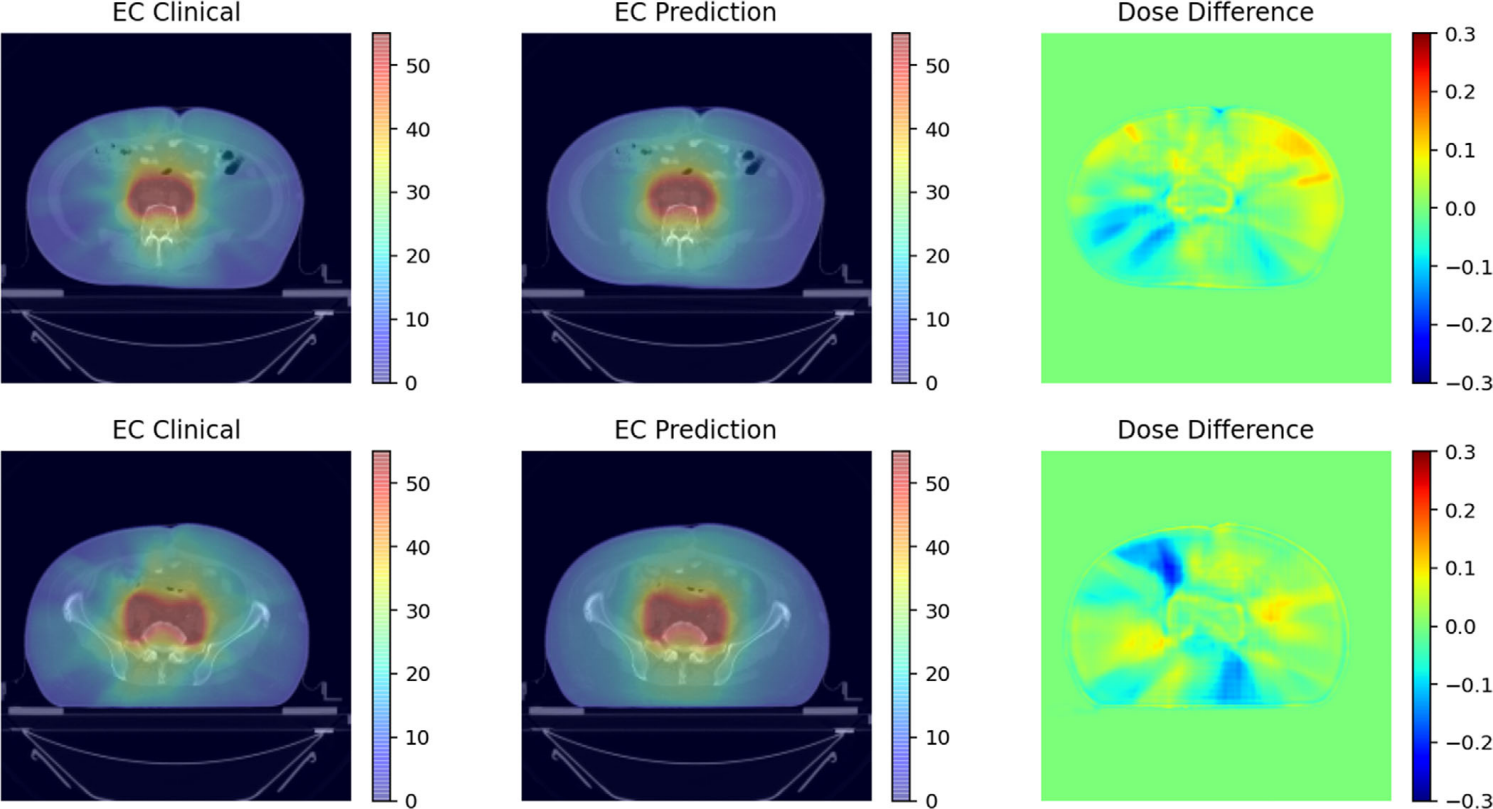
The clinical dose distribution map, the model predicted dose distribution map, and the dose difference map at the same slice of the endometrial cancer case. The left side is the clinical dose distribution map (the unit is Gy), the middle is the predicted dose distribution map, and the right is the dose distribution difference map (take the prescription dose equal to 1 as the standard)

Figures 2 and 3, respectively, show the clinical dose distribution map, predicted dose distribution map, and dose difference map of cervical cancer and endometrial cancer in different trans‐axial slices. It can be seen from the figures that the predicted dose distribution was similar to the clinical dose distribution. It can be seen from Figure 3 that the prediction accuracy of the 35–50 Gy region is high, which may be due to the good consistency of the target and dose distribution in this region for different clinical plans. So, the dose distribution can be accurately predicted. In the low‐dose region (10–35 Gy), due to the various shape and location of OARs for different patients, the dose distribution in the clinical plan was less consistent, which resulted in relatively lower prediction accuracy.

**FIGURE 4:**
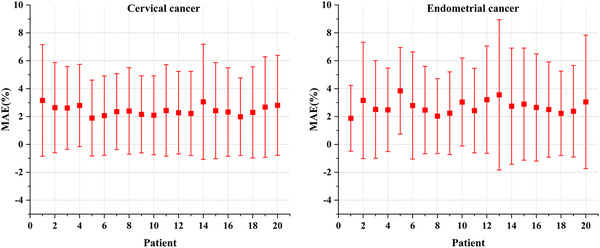
Mean absolute errors of the 3D‐Unet model, including all voxels within the body for the 20 testing cases of cervical cancer and endometrial cancer, respectively

Figures 4 shows the MAE and standard deviation within the body of 20 test cases of cervical cancer and endometrial cancer, respectively. The overall mean MAE and maximum MAE of the cervical cancer cases were 2.43 ± 3.17% and 3.16 ± 4.01% of the prescribed dose and the endometrial cancer cases were 2.70 ± 3.54% and 3.85 ± 3.11%.

### 3.2 Dosimetric indexes and DVHs

**FIGURE 5:**
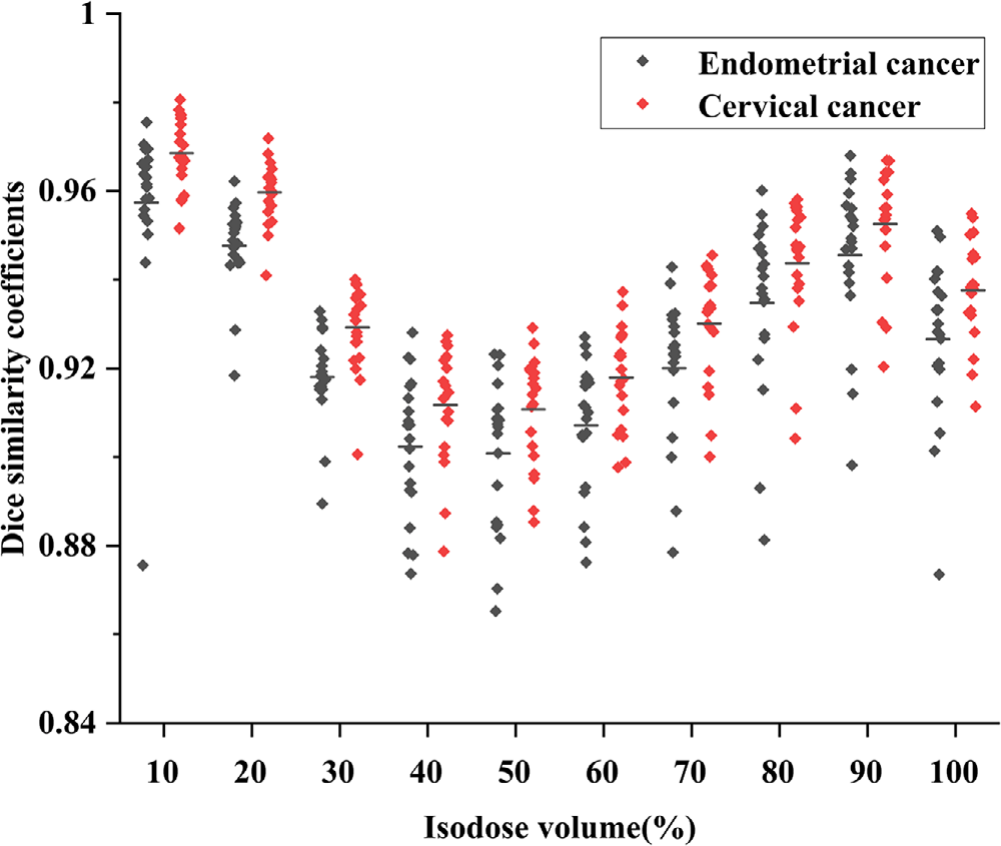
The Dice similarity coefficients (DSCs) of 20 testing cases of cervical cancer and endometrial cancer, respectively, under the isodose volume of 10%–100% (10% interval) of the prescribed dose

**FIGURE 6:**
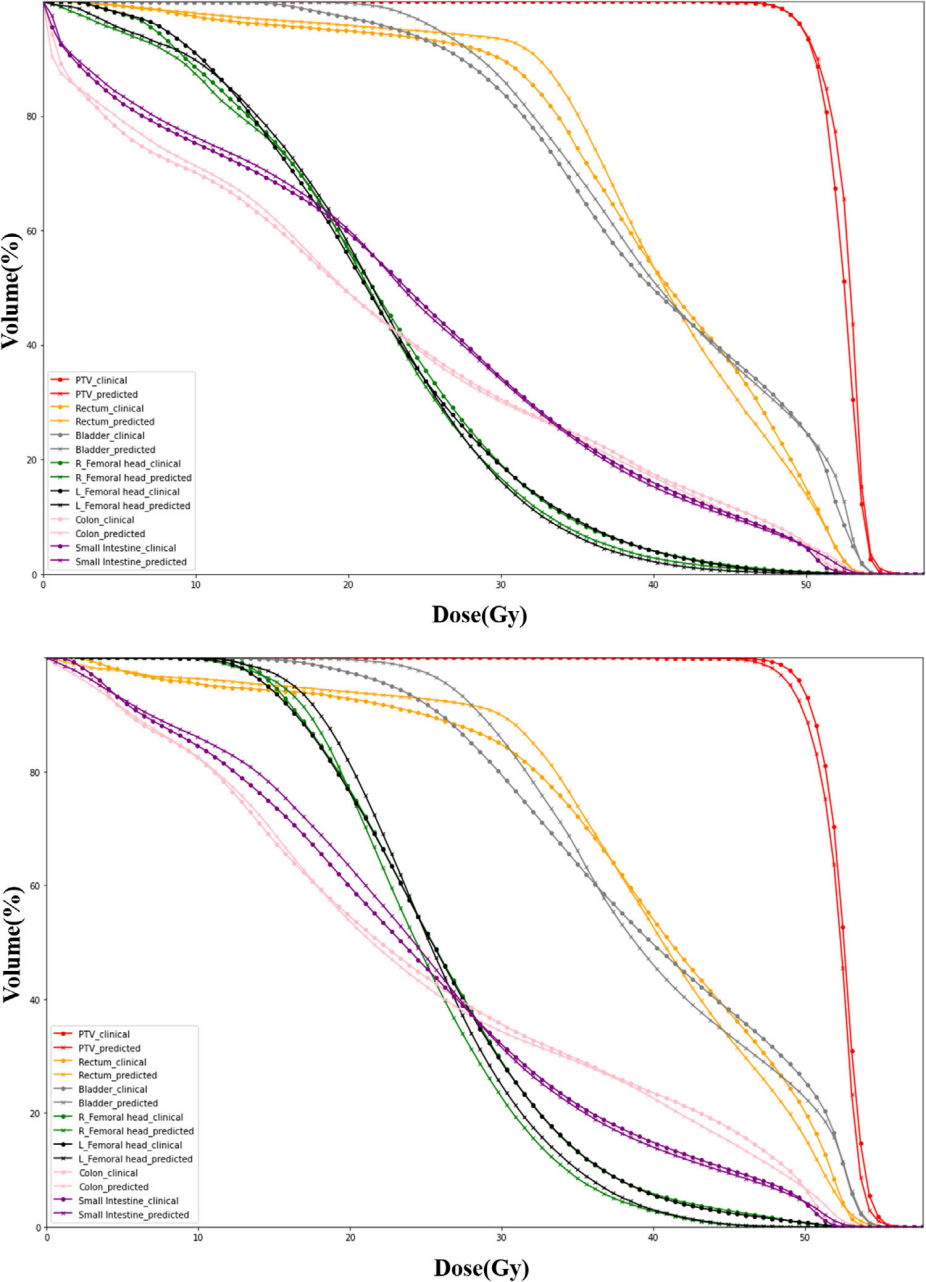
The representative dose–volume histograms (DVHs) of the tested cases. The upper picture shows the result of the cervical cancer case, and the lower picture shows the result of the endometrial cancer case

Table 1 shows the dosimetric indexes between the clinical results and the predicted results of two cancer cases. Figure 5 shows that the average DSCs of cervical cancer and endometrial cancer cases are above 0.9 under the different isodose volume of the prescribed dose, which proves that the 3D‐Unet dose prediction model can accurately predict the dose distribution of endometrial cancer. Figure 6 shows the representative DVHs of cervical cancer and endometrial cancer.

**TABLE 1:** Comparison of dosimetric indexes (mean ± standard deviation)

Dosimetric indexes	CC clinical	CC prediction	*p* ^a^	EC clinical	EC prediction	*p* ^b^
PTV						
HI	0.09 ± 0.02	0.10 ± 0.01	0.126	0.1 ± 0.02	0.12 ± 0.01	0.000
*D*_98%_ (Gy)	48.96 ± 0.49	48.89 ± 0.19	0.263	48.92 ± 0.41	48.27 ± 0.44	0.000
Bladder						
*D*_mean_ (Gy)	40.53 ± 2.84	41.48 ± 1.96	0.067	40.04 ± 2.72	40.36 ± 1.84	0.478
*D*_2cc_ (Gy)	53.50 ± 0.83	53.89 ± 0.46	0.033	53.87 ± 0.67	53.95 ± 0.67	0.494
*V*_40_ (%)	52.20 ± 12.61	54.01 ± 9.21	0.093	50.50 ± 11.72	47.72 ± 7.20	0.370
Colon						
*D*_mean_ (Gy)	23.31 ± 5.25	23.41 ± 5.19	0.911	25.54 ± 3.53	25.16 ± 3.16	0.044
*D*_2cc_ (Gy)	51.62 ± 1.01	52.28 ± 1.05	0.014	51.55 ± 0.60	52.07 ± 0.76	0.004
*V*_40_ (%)	20.07 ± 10.3	19.60 ± 10.02	0.108	24.83 ± 8.68	24.42 ± 8.47	0.370
Femoral head left						
*D*_mean_ (Gy)	23.53 ± 4.73	23.01 ± 4.57	0.126	26.09 ± 3.98	26.06 ± 2.54	0.391
*V*_40_ (%)	4.86 ± 3.67	2.56 ± 2.41	0.000	4.92 ± 8.42	3.11 ± 3.56	0.036
Femoral head right						
*D*_mean_ (Gy)	23.35 ± 4.85	22.59 ± 4.96	0.067	26.08 ± 3.77	25.12 ± 2.65	0.108
*V*_40_ (%)	4.55 ± 4.42	2.95 ± 3.27	0.050	4.86 ± 9.18	2.63 ± 3.68	0.015
Rectum						
*D*_mean_ (Gy)	40.01 ± 3.88	40.51 ± 2.93	0.351	38.91 ± 3.07	39.27 ± 2.76	0.501
*D*_2cc_ (Gy)	51.68 ± 1.08	51.69 ± 1.33	0.911	52.38 ± 0.71	52.43 ± 0.90	0.911
*V*_40_ (%)	55.90 ± 18.02	56.60 ± 13.55	0.526	52.04 ± 9.35	52.29 ± 8.99	0.970
Small intestine						
*D*_mean_ (Gy)	25.39 ± 6.01	25.26 ± 6.14	0.601	24.16 ± 3.67	24.73 ± 4.49	1.000
*D*_2cc_ (Gy)	50.91 ± 2.86	51.13 ± 5.01	0.013	50.99 ± 2.28	51.40 ± 2.42	0.001
*V*_40_ (%)	18.00 ± 10.98	17.00 ± 9.82	0.030	14.35 ± 6.56	14.05 ± 7.16	0.126

*Note*: *D*_mean_ is the average dose, *V*_40_ is the volume of receiving 40 Gy dose, *p* ^a^ is the statistical *p*‐value of cervical cancer clinical and prediction results, and *p* ^b^ is the statistics *p*‐value of endometrial cancer clinical and prediction results.

Abbreviations: CC, cervical cancer; EC, endometrial cancer; HI, homogeneity index; PTV, planning target volume.

## 4 DISCUSSION

To our knowledge, there is no study on cervical cancer VMAT dose prediction and the dose prediction model's generalization between different cancer sites in the previous studies. A 3D‐Unet dose prediction model established using the cervical cancer VMAT plans was used to predict the dose distribution of endometrial cancer in this study. The results were evaluated with the dose distribution map, MAE, DVHs, dosimetric indexes, DSCs, etc. The predicted dose distribution was comparable to the clinical dose distribution, showing the possibility of the generalization of the model. Exploring the generalization of the model helps to expand the application scope of the model, and it is not necessary to build a model based on every cancer site again, which reduces the data collection work before the model is established. In this study, there is no need to collect a large number of endometrial cancer cases for modeling again, and the dose prediction model for cervical cancer can be used to predict the dose distribution for endometrial cancer accurately. For some small to medium institutions, this is extremely important, because it is difficult or impossible for them to collect enough cases to establish high‐quality dose prediction models for different cancers. Therefore, it is of great significance to explore the generalization of the dose distribution prediction model with a relatively large number of cancer cases to a relatively small number of other cancer cases.

In this study, the cervical cancer dose prediction model can predict the dose distribution for endometrial cancer cases accurately, suggesting the possibility of model generalization. The reason we consider the successful generalization of the model is that both cervical cancer and endometrial cancer are postoperative cases, and the OARs that need to be protected are the same, mainly the bladder, left and right femoral heads, colon, rectum, and small intestine. The same delivery technique of VMAT and the prescribed doses of 50.4 Gy were used for both sites. Also, the dose distributions were similar for both sites. Therefore, the model can be generalized from cervical cancer to endometrial cancer. Kandalan et al.^24^ explored the generalization of the dose prediction model for prostate cancer treated with VMAT. The possibility of generalization between different dose distribution styles of the same cancer site was investigated. Different styles of cases were added to the source model to improve the generalization performance.

There are some limitations to this study. Firstly, the results initially show the feasibility of model generalization on different cancer sites. Although the dose distribution is clinically acceptable for endometrial cancer, it is not optimal, which may be caused by the structure difference between cervical cancer and endometrial cancer. The PTV volume of cervical cancer was larger than that of endometrial cancer, and the distance between PTV and OAR of cervical cancer was greater than that of endometrial cancer, which made the predicted dose of OARs of endometrial cancer higher than the clinical dose distribution. Some cases of endometrial cancer could be added to the training set to further improve the generalization performance of the model in future studies. Secondly, to accelerate the training process, we first down‐sampled the slice of 3D volume to 256 × 256, and then clip the data to 128 × 128. The clipped data contain main information without enormous unnecessary voxel. During testing phase, we can apply data with slice size of 256 × 256 or 512 × 512 since our model is a fully convolutional network. However, this clip operation may miss some information of 3D volume which may degrade the model performance. Thirdly, it is difficult to predict a personalized dose distribution for a specific patient using this model. For example, in clinical practice, clinicians may prefer to preserve the patient's rectum or bladder, and the model cannot create such a specific plan for rectum sparing or bladder sparing. In future work, the dose distribution of some specific plans such as rectum sparing or bladder sparing plans should be included to create a specific deep learning model to meet certain preferences. The maximum standard deviation of MAE was about 3% and 5% for only a few cervical cancer and endometrial cancer patients and less than 3% for the majority of the tested patients for both cervical and endometrial cancer, which is similar or better than previous studies. The big error mainly occurred in the cases with different target and OARs configuration from the majority cases. In addition, the contour variations within or between institutions may also influence the model performance, which is a future work we will consider.

## 5 CONCLUSION

A deep learning dose prediction model based on 3D‐Unet for cervical cancer VMAT plans was developed and evaluated. The model can accurately predict the clinical dose distribution for cervical cancer. It can also be generalized to endometrial cancer cases with equivalent performance.

## AUTHOR CONTRIBUTIONS

*Model design, training, testing, data collection, analysis, paper writing, and revision*: Zhang Qilin and Bao Peng. *Data preparation*: Qu Ang, Jiang Weijuan, and Jiang Ping.

*Study design, paper writing, and revision*: Zhuang Hongqing, Dong Bin, and Yang Ruijie.

## CONFLICT OF INTEREST

The authors have no conflicts of interest to disclose.
